# Catheterism

**Published:** 1844-07-13

**Authors:** 


CATHETERISM.

Sir Astley Cooper says that when it is desired to introduce a catheter into the bladder during the existence of enlargement of the prostate gland, an instrument of elastic gum may be selected, which being introduced as far as the obstruction caused by the enlarged gland, the stilette may be partly withdrawn, by which process the point of the catheter will be raised, and then may be pushed on into the bladder. This, Mr. Chippendale says, is a very useful hint, but we would add, as a caution to young practitioners, when the stilette has been thus partially withdrawn, it should not be returned while the instrument is in the passage, for if the gland is only laterally enlarged, the point of the catheter may be so diverted from the straight line, that the eye may allow the stilette to pass outside, and thus lacerate the parts. This happened to him some time ago, and fortunately so, for the point of the stilette punctured an abscess, of the existence of which he had not any suspicion; a large quantity of pus was discharged, and the patient recovered. But it is not every case which might terminate so favourably.—*Lond. Med. Times.*

